# Viral Zoonoses: Interactions and Factors Driving Virus Transmission

**DOI:** 10.3390/v16010009

**Published:** 2023-12-20

**Authors:** Myriam Ermonval, Serge Morand

**Affiliations:** 1 Unité Environnement et Risques Infectieux, Institut Pasteur, Université Paris-Cité, 75015 Paris, France; 2 IRL2021 HealthDEEP (Health, Disease Ecology, Environment and Policy), CNRS (Centre National de la Recherche Scientifique)–Kasetsart University–Mahidol University, Bangkok 10900, Thailand; 3 Faculty of Veterinary Technology, Kasetsart University, Bangkok 10900, Thailand

## 1. Introduction

The beginning of the 21st century was marked by an increase in the number of emerging/reemerging infectious diseases detected worldwide and by the challenging COVID-19 pandemic. Most of these emerging diseases are caused by viruses that are primarily RNA viruses of animal origin, with a long history of adaptation to their natural hosts, becoming pathogenic when crossing species barriers [1]. In humans, they can cause serious illness that can be sometimes fatal, with some virus species having a high mortality rate in the groups of neuropathies (Nipah virus, Rabies virus, etc.), of acute pulmonary syndromes (SARS-CoV-2, Influenza viruses), or of viral hemorrhagic fevers, as exemplified by some species of *Bunyavirales* (hantavirus, arenavirus), *Flaviviridae* (Dengue virus), and *Filoviridae* (Ebola virus) [2]. The conditions for viral persistence in animal reservoirs, particularly among the very diverse species of rodents and bats [3], and pathogenicity in humans are not always elucidated [4]. Meanwhile, outbreaks are influenced by human activities that disrupt ecosystems and increase contact between infected animals and humans. Therefore, population dynamics are of great importance, with domestic animals playing key roles as intermediate hosts in the transmission of viruses [5,6]. Determining interactions at different levels (molecular, cellular, systemic) of viruses with their different human and animal hosts [7,8,9] and their persistence in the environment [10] is of great importance for assessing transmission risks. Above all, the contribution of different disciplinary approaches integrated into the “One Health” concept is now recognized as being fundamental for the implementation of measures to prevent new viral emergences [11]. In this context, the spillover to other species, even from humans to domestic or wild animals in the event of a major epidemic episode, as recently demonstrated by the spillback of SARS-CoV-2 to farmed minks [12], white-tailed deer [13], and rodents [14,15], constitutes a major risk to be considered.

## 2. Contents

The contributions to this Special Issue on Viral Zoonoses cover general aspects of the pathophysiology of zoonotic viruses in different hosts and assess the role of certain factors favoring their transmission, therefore, as reviewed in Agusi et al. 2022, addressing the importance of One Health approaches, including a broad community of scientists working in different fields.

The published studies highlight the requirement for:

(i). Good surveillance (epidemiology) of the viruses present in wild and domestic animals: Hamel et al. 2023 discovered a new flavivirus infecting mosquitoes in rural areas of Thailand. The circulation of the highly pathogenic H5N1 avian flu virus responsible for sporadic outbreaks is presented in a seroprevalence and meta-analysis study carried out by Ntakiyisumba et al. 2023, supporting the necessity of monitoring the avian influenza virus to protect farm birds from contamination.
(ii). Pertinent techniques to identify and diagnose viruses and their possible transmission to humans: To obtain insights into inter-host adaptation, Embregts et al. 2022 used NGS to analyze virus populations within specific hosts and tissues by isolating the Rabies virus from a broad range of CNS and non-CNS samples of mouse and human origin. The comparative study of the different detection assays used to detect Nipah virus infection, as reviewed by Garbuglia et al. 2023, illustrates the importance of high sensitivity tests for the management of epidemics.
(iii). Ecological and anthropological approaches to assess the risk and consequences of epidemics: The influence of climate change on outbreaks due to henipaviruses circulating in bats was evaluated in an article by Latinne and Morand 2022. A study conducted by Rojas Sereno et al. 2022 exploring the factors associated with the spatial expansion of bats carrying the Rabies virus in Colombia shows the importance of these data for reducing epidemics by improving vaccination.

Furthermore, understanding the transmission factors between different animal species, for example, between wild and domestic animals, through spillover between different animal species or from animals to humans, as well as from humans to farm animals, is essential to predict and limit the risk of new pandemics. Such questions are addressed in some of the publications in the Special Issue: the implication of contact between wild and domestic animals and the increasing risk of emergence is shown by the work of Morcatty et al. 2022 carried out to identify viruses in different wild animals close to domestic ones on sale in Indonesian wildlife markets. Virus transmission between animal species and possible spillback from humans to animals are examined in the studies of Souza et al. 2023 and Kimble et al. 2022 that describe the circulation of influenza viruses in farm pigs, and in the study carried out by Vandegrift et al. 2022 on SARS-CoV-2 found in white-tailed deer.

## 3. Conclusions

Faced with the increase in emerging infectious diseases, their occurrence on a global scale, and the damage caused to ecosystems, mainly by human activities, thereby increasing the contact between wild animals, domestic animals, and humans, the global “One Health” approach is essential. The articles published in the Special Issue “Viral Zoonoses: Interactions and Factors Driving Virus Transmission” contribute to this reflection.
